# A novel bovine viral vaccine for reproductive safety and performance in cattle

**DOI:** 10.3389/fvets.2026.1842435

**Published:** 2026-07-01

**Authors:** Marta Gibert, Carlos Montbrau, Mariona Tapiolas, Héctor Santo-Tomás, Christopher Chase

**Affiliations:** 1 HIPRA S.A., Amer (Girona), Spain; 2 Department of Veterinary and Biomedical Sciences, South Dakota State University, Brookings, SD, United States

**Keywords:** BoHV1, BVDV, cattle, conception, safety, latency, marker, vaccine

## Abstract

Bovine herpesvirus-1 (BoHV-1), the causative agent of infectious bovine rhinotracheitis (IBR), and bovine viral diarrhea virus (BVDV) vaccines are one of the tools that can be used to improve reproductive efficiency, but ordinary modified-live BoHV-1 components can establish latency, impair fertility or cause abortion. This work evaluated the reproductive safety of a gene-deleted BoHV-1 (gE−/tk−) marker strain used alone or within a multivalent vaccine (BoHV-1 gE−/tk−, recombinant BVDV-1 and BVDV-2 E2 protein, modified live bovine respiratory syncytial virus, inactivated parainfluenza-3). Latency and reactivation were examined in 10 seronegative calves vaccinated twice and later treated with dexamethasone, with non-vaccinated calves and virulent BoHV-1 infection included as negative and positive controls, respectively. Conception effects were assessed in four preclinical heifer trials including vaccinated and non-vaccinated control groups, differing in age and vaccination-to-breeding interval, as well as in two gestational safety studies conducted at different trimesters using either the commercial antigen dose (1X) or a 10-fold increase (10X) of the IBR antigen content. A large field trial on four commercial dairies evaluated conception rate and pregnancy loss in vaccinated animals compared with controls. Vaccine virus was not recovered from nasal swabs, trigeminal ganglia, or tonsils after dexamethasone, whereas challenge calves showed typical reactivation and shedding. Across preclinical trials, vaccination did not reduce conception rate. In both 1X and 10X gestation studies, no abortions occurred, and all viable calves were clinically normal and BoHV-1 seronegative at birth. In the field trial, a farm with extensive records demonstrated a conception rate significantly higher in the vaccinated animals after the second dose compared to controls, while similar rates were observed following the other doses. Pregnancy loss did not differ between vaccinated and control animals. These data demonstrate that the gE−/tk− BoHV-1 marker strain, alone or in a multivalent format, does not establish detectable latency or adversely affect fertility or gestational outcome, supporting its reproductive safety in cattle worldwide.

## 1 Introduction

Reproductive efficiency is essential for any profitable cattle enterprise, and preventive vaccination represents an important component of reproductive performance programs. For cattle vaccines, efficacy and reproductive safety against the two major viral reproductive pathogens, bovine herpesvirus 1 (BoHV-1) and bovine viral diarrhea virus (BVDV), are critical. Both viruses are significant contributors to reproductive failure in cattle, causing estrus delay, reduced conception rates, and abortions (1). BoHV-1, the causative agent of infectious bovine rhinotracheitis (IBR), can establish latency and recrudesce during stress or breeding, directly impairing reproductive performance. BVDV, with its immune dysfunction effects, further compromises fertility by disrupting early embryonic development and placental function (1). Vaccines against these pathogens are essential for controlling disease transmission and improving herd productivity, but their safety must be rigorously assessed, particularly in breeding females (1). When referring to BVDV or BoHV-1, ordinary modified live virus (MLV) vaccines, while effective at inducing protective immunity, carry a risk of transient reproductive effects if administered improperly or too close to breeding, and can cause latency (2–5). These effects may include suppression of estrus behavior, delayed ovulation, lower conception rates, or early embryonic loss (2, 3, 5). Killed virus vaccines do not affect conception rate (2) and are safe for pregnant cattle but may provide less robust protection (1). Therefore, ensuring reproductive safety through proper vaccine selection, timing of administration, and understanding of vaccine-virus interactions is vital for optimizing reproductive outcomes, preventing fetal loss, and maintaining overall herd health and economic viability in beef and dairy production systems.

Combination of BVDV and BoHV-1 vaccines are standard tools for reproductive management. The BoHV-1 MLV fraction is associated with vaccine safety issues (4). Ordinary attenuated MLV BoHV-1 establishes latency (6), affects conception, and can induce abortion in naïve or poorly vaccinated animals (4, 6). Thymidine kinase (tk−) mutant BoHV-1 is less virulent than tk+ strains, less likely to establish latency, and protects against infection by virulent field strains (7, 8). BoHV-1, containing a deletion in gE− (9), protected against experimental infection, and the gE− virus was not isolated after treatment with dexamethasone. The gE− strains of BoHV-1 are attenuated and immunogenic and can be used to differentiate infected from vaccinated animals (DIVA). They do result in latency but less recrudescence when reactivated. In a first study (10), the virulence and immunogenicity of three BoHV-1 deletion strains –gE−, tk−, and gE−/tk− (double deletion)– were compared. After intranasal inoculation, the tk− strain had attenuated virulence, while the gE− and gE−/tk− strains were avirulent. The three mutants protected calves against an experimental BoHV-1 infection (10). In a subsequent study, none of the three mutants were recovered after calves were treated with dexamethasone (9). A novel multivalent vaccine containing modified live, inactivated, and subunit components has been developed. The vaccine combines a genetically modified live BoHV-1 (gE−/tk−), recombinant E2 glycoproteins of BVDV-1 and BVDV-2, a live attenuated bovine respiratory syncytial virus (BRSV), and inactivated bovine parainfluenza 3 (PI3) virus. This formulation has demonstrated respiratory efficacy (11, 12) and reproductive protection (13). Further studies indicate that the same gE−/tk− BoHV-1 strain has yielded positive outcomes in enhancing reproductive performance and in limiting BoHV-1 circulation, as evidenced by reduced seroprevalence across herds (14, 15).

This study complements previous findings by evaluating the safety of both the monovalent modified BoHV-1 (gE−/tk−) vaccine and the multivalent combination vaccine, with particular emphasis on their effects on reproductive outcomes in cattle.

## 2 Materials and methods

### 2.1 Vaccines

Three vaccines were used: (i) a monovalent modified live BoHV-1 (gE−/tk−) vaccine at the commercial antigen dose (HIPRABOVIS® IBR Marker Live Vaccine, HIPRA); (ii) a monovalent vaccine containing a 10-fold (10X) increase of the same modified live BoHV-1 (gE−/tk−) antigen; and (iii) a multivalent vaccine (DIVENCE® PENTA, HIPRA) containing the same modified live BoHV-1 (gE−/tk−) antigen at the commercial dose. The multivalent vaccine also contained live attenuated BRSV, inactivated PI3 virus, and recombinant E2 glycoproteins of BVDV-1 and BVDV-2. The vaccines were reconstituted and diluted according to the manufacturer’s instructions.

### 2.2 Reproductive safety studies

Four independent studies were conducted to evaluate the reproductive safety of the vaccine. Three preclinical studies (Studies 1–3) were performed at the HIPRA Research Facilities in Amer, Spain, and one multicenter field study (Study 4) was conducted at four commercial dairy farms, three in Spain and one in Hungary.

All studies were conducted under randomized and blind conditions. Personnel involved in clinical evaluation and data collection were unaware of treatment allocation. Vaccine administration (Treatment Dispenser) was performed by a designated individual not involved in outcome assessment, following a treatment allocation key generated by the Study Director.

#### 2.2.1 Study 1: BoHV-1 latency reactivation and re-excretion after vaccination and dexamethasone treatment

**Figure 1:**
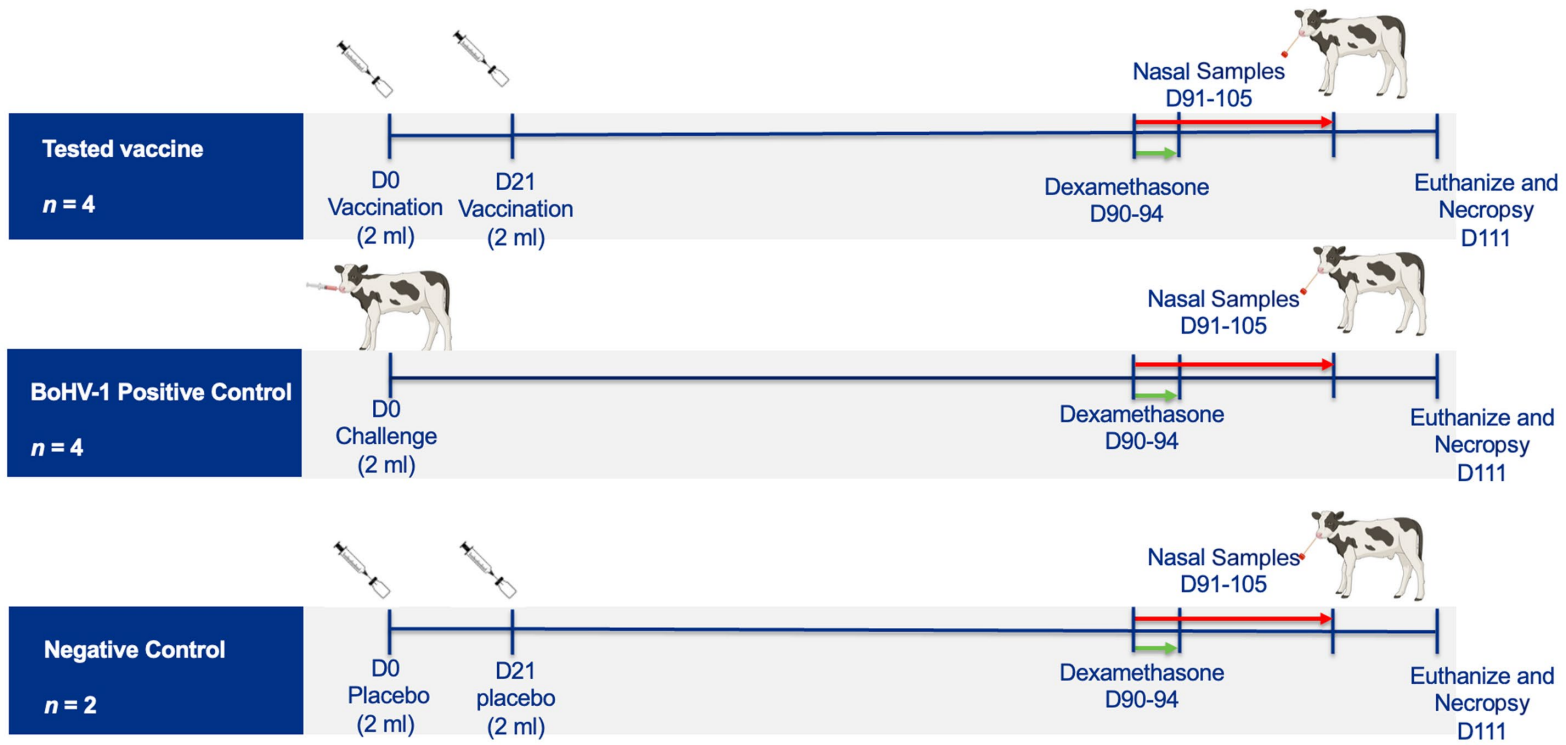
Recrudescence with the monovalent modified BoHV-1 (gE−/tk−) vaccine.

In this study, 10 Holstein-Friesian BoHV-1 seronegative calves, approximately 3 months of age at the time of vaccination, were used. Four seronegative calves were vaccinated intramuscularly (IM) with the monovalent modified BoHV-1 (gE−/tk−) vaccine and revaccinated 21 days later IM (2 mL/dose) at the maximum vaccine dose (10^7.3^ CCID50/calf). Two calves were kept as negative, unvaccinated control animals. The vaccinated and negative-control calves were housed together for 90 days. Four positive control calves were inoculated with 10^8.3^ CCID50/calf of a virulent BoHV-1 Iowa challenge strain intranasally and housed separately until study day 91 (Figure 1). Clinical observations for adverse events were performed before vaccine administration, challenge, or dexamethasone treatment, and 3 h after each. General health observations were done daily. Blood samples were collected for BoHV-1 serology at D0 and Day 91 of the study. Three months (90 days) after vaccination, all calves were housed together and treated with dexamethasone (0.1 mg/kg IM for 5 consecutive days). Latency, reactivation, and re-excretion of the vaccine virus were analyzed over a 15-day follow-up period (Figure 1). Viral excretion was determined by collecting nasal swab samples, followed by isolation and identification of BoHV-1 by viral titration (16). Samples in which the IBR virus was isolated were also analyzed by PCR to demonstrate that the IBR virus identified was either a field virus or a vaccine virus. The animals were euthanized at the end of the 21-day reactivation period, and palatine and pharyngeal tonsils, as well as trigeminal ganglia, were collected at necropsy and then processed for virus isolation (17).

#### 2.2.2 Study 2: effect of vaccination timing on conception rate

**Figure 2:**
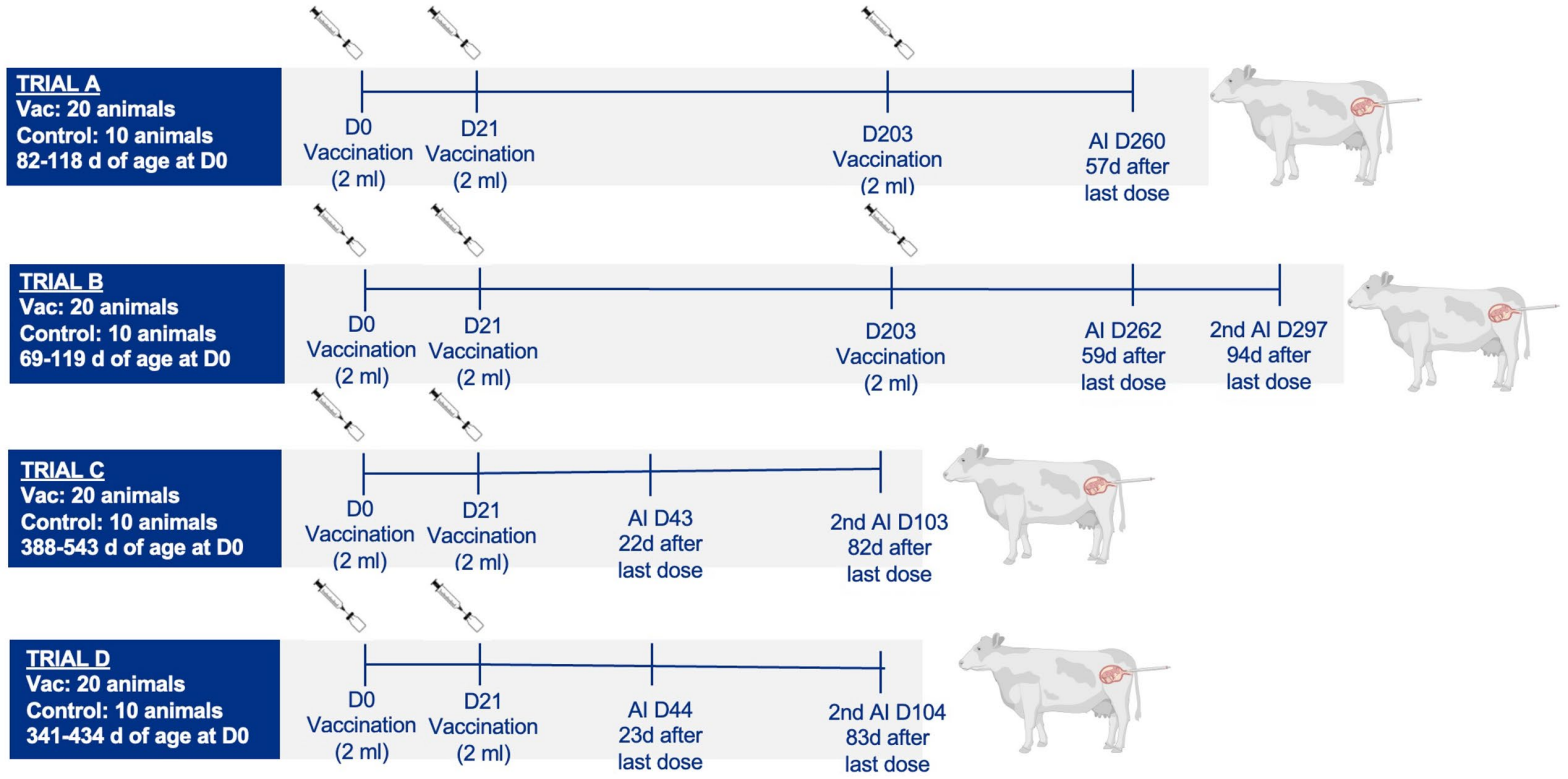
The effect of vaccination on conception.

The studies were conducted in two different age groups (Figure 2).

##### 2.2.2.1 Heifer calves (trials A and B)

Sixty BoHV-1 and BVDV seronegative, multiple-source heifers of two to 3 months of age (most of them Holstein-Friesian) were enrolled. Forty animals (divided into two groups of 20 heifers each) were vaccinated with the multivalent vaccine, and 20 animals (two groups of 10 heifers) served as controls and were sham vaccinated with phosphate buffered saline (PBS). The animals (20 vaccinates and 10 controls for each Trial) were divided equally between Trials A and B with the only difference in design being a single artificially inseminated (AI) breeding in Trial A vs. two AI breedings in Trial B (Figure 2). All animals were vaccinated at ~90 days of age (range: 70–120 days) and revaccinated 21 days later (Figure 2). The animals were then revaccinated a third time ~6 months later (~180 days). Clinical observations for adverse events were performed before vaccine administration and 3 h after vaccination. General health observations were done daily. Heifers were synchronized at approximately 12 months of age (2–3 months after the booster); they were given 1 mL IM gonadotropin-releasing hormone (GnRH, Gestavet-GnRH, HIPRA), and an intravaginal progesterone-impregnated controlled internal drug release device (CIDR, Zoetis) was inserted. The CIDR devices were removed after 5 days, and 1 mL of prostaglandin was administered to the heifers (D-cloprostenol, Gestavet-Prost, HIPRA). A second dose of prostaglandin was administered 24 h later. Forty-eight hours later, the heifers were given 1 mL IM GnRH and were AI using frozen semen from a BVDV-free bull. Heifers were confirmed to be pregnant by transrectal ultrasonography approximately 35 and 90 days after artificial insemination. In trial B, open heifers were synchronized and AI as described above, and AI bred a second time ~2 months (~60 days; 2 months interval) later. Pregnancy rates and the number of AI services were measured at 70–75 days following breeding by ultrasound.

##### 2.2.2.2 Yearling heifers (trials C and D)

Sixty BVDV seronegative Holstein-Friesian heifers, 12-to-18 months of age were enrolled. Forty animals (divided into two groups of 20 heifers each) were vaccinated with the multivalent vaccine, and 20 animals (two groups of 10 heifers) served as controls and were sham vaccinated with PBS. All animals were vaccinated at >1 year of age, and revaccinated 21 days later (Figure 2). Clinical observations for adverse events were performed before vaccine administration and 3 h after vaccination. General health observations were done daily. The animals were synchronized and bred by AI 22–23 days after the last vaccination as described above. In trials C and D, the open animals were synchronized, and AI bred a second time ~2 months (~60 days: 2 months interval) later. Pregnancy rates and the number of AI services were measured using ultrasound at 70–75 days.

#### 2.2.3 Study 3: effect of vaccination of animals during gestation

##### 2.2.3.1 Trial 1—effect of vaccination with the field vaccine dose during gestation

**Figure 3:**
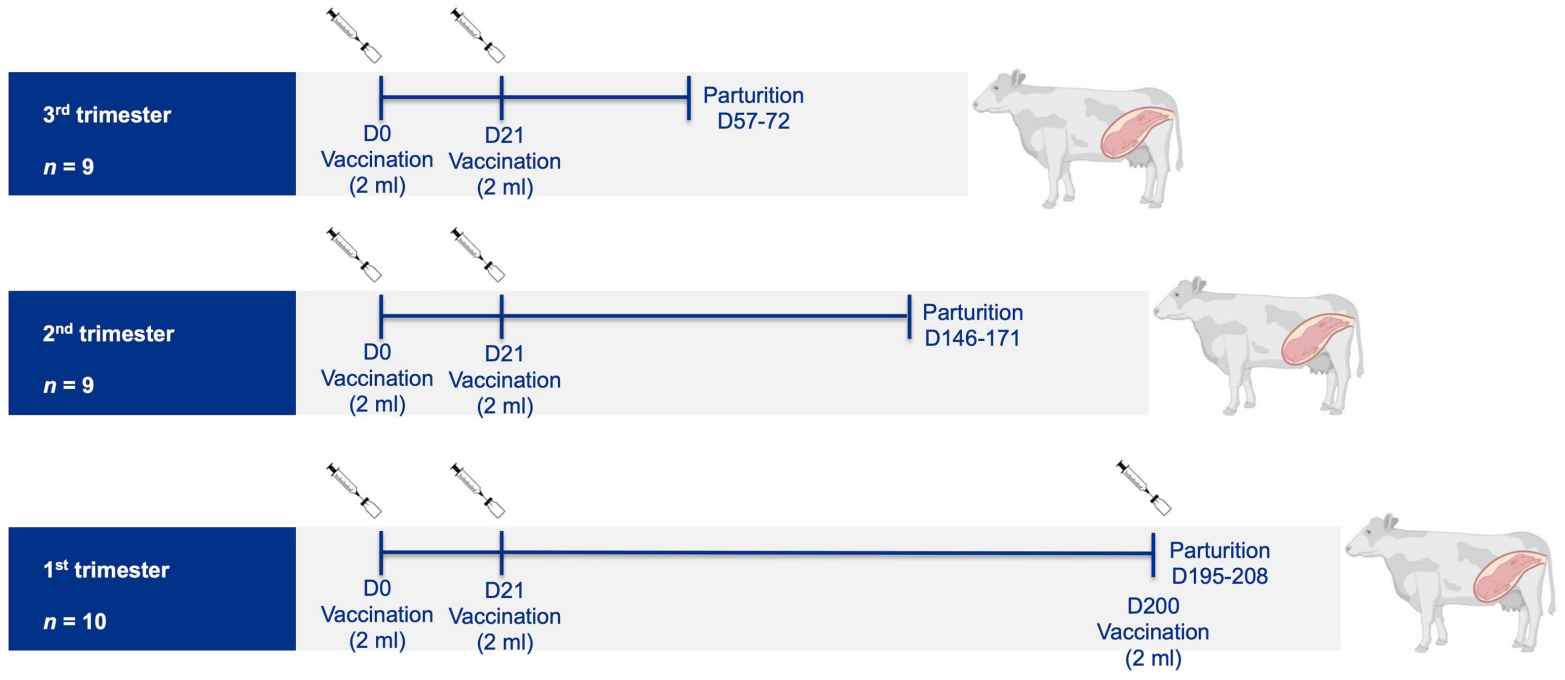
Pregnant heifers vaccinated with field vaccine dose.

Cows: Twenty-eight BoHV-1 and BVDV seronegative Holstein-Friesian cows (three groups of ~9 or 10 animals per gestation length group) were enrolled (Figure 3). The animals at three stages of gestation: first trimester (~78 days of gestation-10 animals); second trimester (~122 days of gestation-9 animals); or third trimester (~208 days of gestation-9 animals) received a priming IM dose of the multivalent vaccine at the field dosage, with a total titer of 10^7.6^ TCID50. The pregnant animals were all revaccinated 21 days later with the vaccine IM at the field dosage. The first-trimester group received a third IM vaccination 5–7 days before calving, at the field dosage. General health observations were done twice daily on the cows until they calved. Clinical observations for adverse events were performed before vaccine administration and 3 h after vaccination. Blood was taken for BoHV-1 serology on D0.

Calves: Calf health was assessed twice a day.

##### 2.2.3.2 Trial 2—effect of vaccination with a 10-fold (10X) increase of the IBR antigen content during pregnancy

**Figure 4:**
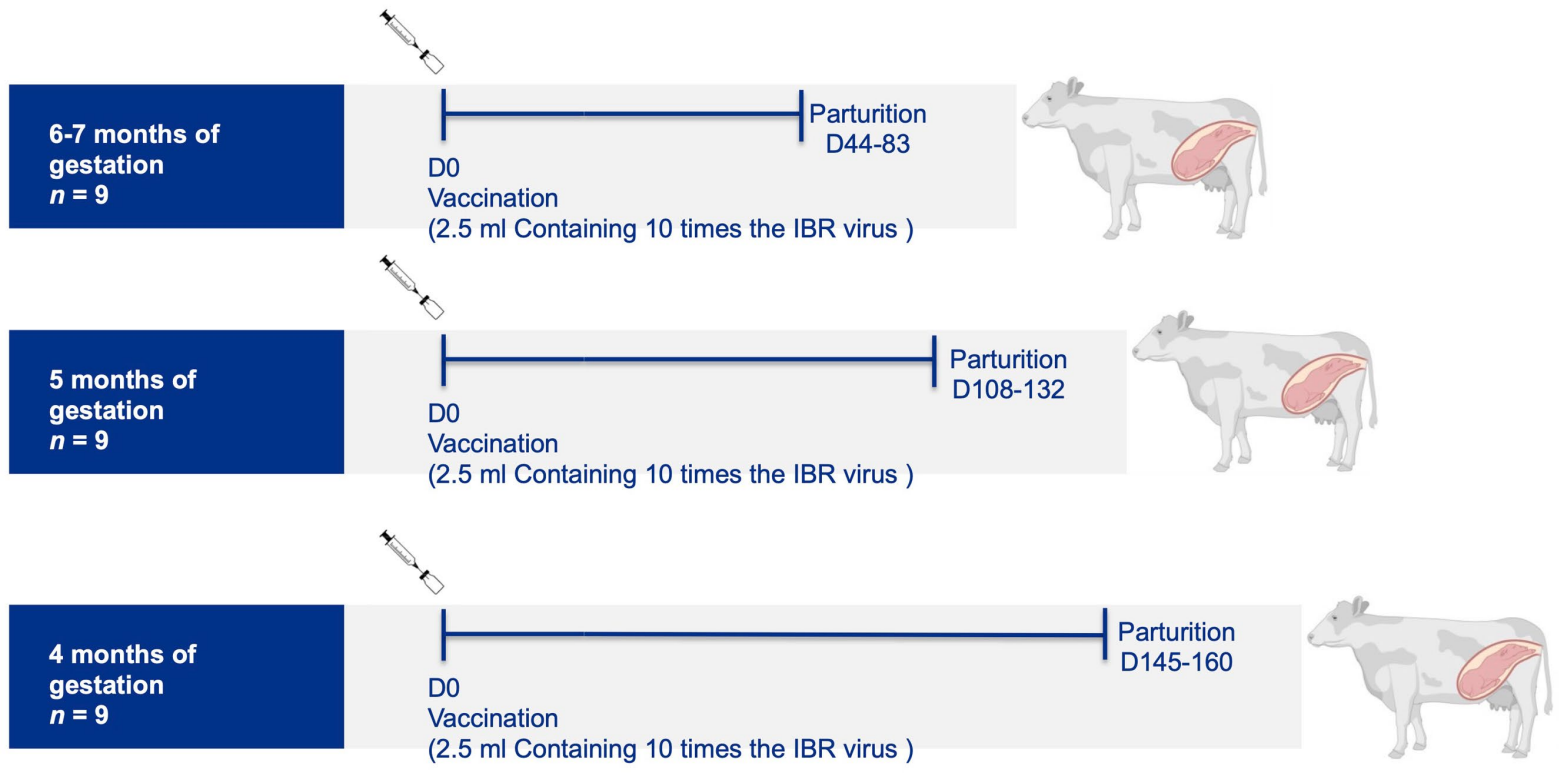
Pregnant vaccinated with a 10-fold (10X) increase of the IBR antigen content.

Cows: Twenty-seven BoHV-1 seronegative Holstein-Friesian cows (three groups of 9 animals per gestation length group) were enrolled (Figure 4). The animals were vaccinated at one of three gestational stages in the study: 4 months (9 animals), 5 months (9 animals), or 6–7 months (9 animals). Those animals were purchased from commercial farms, and nasal swabs were collected 2 days after arrival and tested for BoHV-1 by virus isolation and real-time-PCR. The PCR assay was performed using TaqMan probe chemistry with BoHV-1-specific primers and a hydrolysis probe targeting a conserved region of the viral genome. Primer and probe sequences were as follows: forward primer gB-F (5′-TGT GGA CCT AAA CCT CAC GGT-3′), reverse primer gB-R (5′-GTA GTC GAG CAG ACC CGT GTC-3′), and probe gB (5′-FAM-AGG ACC GCG AGT TCT TGC CGC-TAMRA-3′). The animals were vaccinated with a single IM dose of the monovalent vaccine containing a 10-fold (10X) increase of the IBR antigen content relative to the commercial formulation (4 mL; total titer 10^8.6^ TCID50). Clinical observations for adverse events were performed before vaccine administration and 3 h after vaccination. General health observations were done twice daily on the cows until they calved. Blood was collected for BoHV-1 serology on D0 and at parturition and tested by ELISA (CIVTEST® BOVIS antibody test, HIPRA) to detect BoHV-1 antibodies.

Calves: Calf health was assessed daily through day 3, and BoHV-1 antibody titers were measured prior to colostral intake.

#### 2.2.4 Study 4: Field study of the effect of vaccination on reproductive safety

##### 2.2.4.1 Study design

The study was carried out between July 2021 and September 2023 at four commercial dairy farms. Three of the farms were located in Spain (Farm 1 and Farm 2 in Balearic Islands, and Farm 3 in Catalonia), and the fourth farm, was in Hungary (Table 1). A total of 1,255 (pregnant, recently inseminated, and nonpregnant) healthy Holstein-Friesian females, from 10 weeks of age onwards, were initially included in the clinical study and randomly assigned to vaccine and control (PBS) groups (Table 1). The animals were blocked by parity, lactation status (lactating or non-lactating; Table 2), and stage of pregnancy (Table 3). All the animals from the same farm and belonging to the vaccine group were vaccinated on the same day (whole herd vaccination). Animals were at varying stages of heifer development, lactation, or gestation status on each vaccination day and were vaccinated four times (Figure 5). The first dose was administered to 1,255 animals, the second dose was administered to 1,238 animals, the third dose was administered to 1,071, and the fourth dose was administered to 826 (Tables 2, 3). The first dose was given on enrollment, the second dose (3 weeks after the first dose) ~21 days later; the third dose (6 months after the second dose) ~203 days after the first dose, and the fourth dose (Booster, one year after the third dose) ~568 days after the first dose (Figure 5). The subsequent doses (2, 3, and 4) were administered to the remaining animals in the study, as animals were culled as part of standard management (Table 1). Over the 1-year period between the third and fourth doses, multiple animals had been culled under standard management and removed from the study (Tables 2, 3).

**Table 1:** Distribution of cattle in the four reproduction field studies at enrollment.

Group	Category	Distribution for Cows/Heifers	Stratum	Farm				Total
				Farm 1	Farm 2	Farm 3	Farm 4^#^	
Control	Cow	Parity^*^	1	50	13	67	n.a	130
			2	35	14	40		89
			3	28	9	21		58
			4	13	3	7		23
			>4	16	4	8		28
		Total cow		142	43	143		328
	Heifer	Age (months)	≥12	67	22	31	0	120
			<12	51	8	17	100	176
		Total heifer		118	30	48	100	296
		Total control		260	73	191	100	624
Vaccine	Cow	Parity^*^	1	60	16	67	n.a	143
			2	35	11	45		91
			3	24	10	22		56
			4	11	3	9		23
			>4	16	3	4		23
		Total cow		146	43	147		336
	Heifer	Age (months)	≥12	61	22	32	0	115
			<12	51	10	19	100	180
		Total heifer		112	32	51	100	295
		Total vaccine		258	75	198	100	631

n.a: not applicable.

* The study population was grouped based on parity (lactation number).

# Only heifers were included from farm 4.

**Table 2:** Enrollment of lactating vs. non-lactating in the field safety study.

Vaccination dose	Group	Lactating	Non-lactating^#^	Total
1st dose	Control	290	334	1,255
	Vaccine	296	335	
2nd dose	Control^*^	282	333	1,238
	Vaccine	291	332	
3rd dose	Control^*^	282	244	1,071
	Vaccine	296	249	
Booster dose	Control^*^	305	112	826
	Vaccine	299	110	

* Data for all of the study animals was not available.

# Dry cows and heifers.

**Table 3:** Enrollment of pregnant vs. non-pregnant in the field safety study.

Vaccination dose	Group	Pregnant				Recently inseminated^#^	Non-pregnant	Total animals
		1T	2T	3T	Total pregnant			
1st dose	Control	60	106	64	230	86	308	1,255
	Vaccine	58	106	70	234	88	309	
2nd dose	Control^*^	61	102	60	223	67	325	1,238
	Vaccine	58	104	70	232	77	314	
3rd dose	Control^*^	83	52	45	180	81	265	1,071
	Vaccine	74	64	43	181	72	292	
Booster dose	Control^*^	63	41	96	200	83	133	826
	Vaccine	52	54	85	191	73	147	

1 T: 1st trimester; 2 T: 2nd trimester; 3 T: 3rd trimester.

* Data for all of the study animals was not available.

# Recently inseminated, animals inseminated but not confirmed pregnancy.

**Figure 5:**
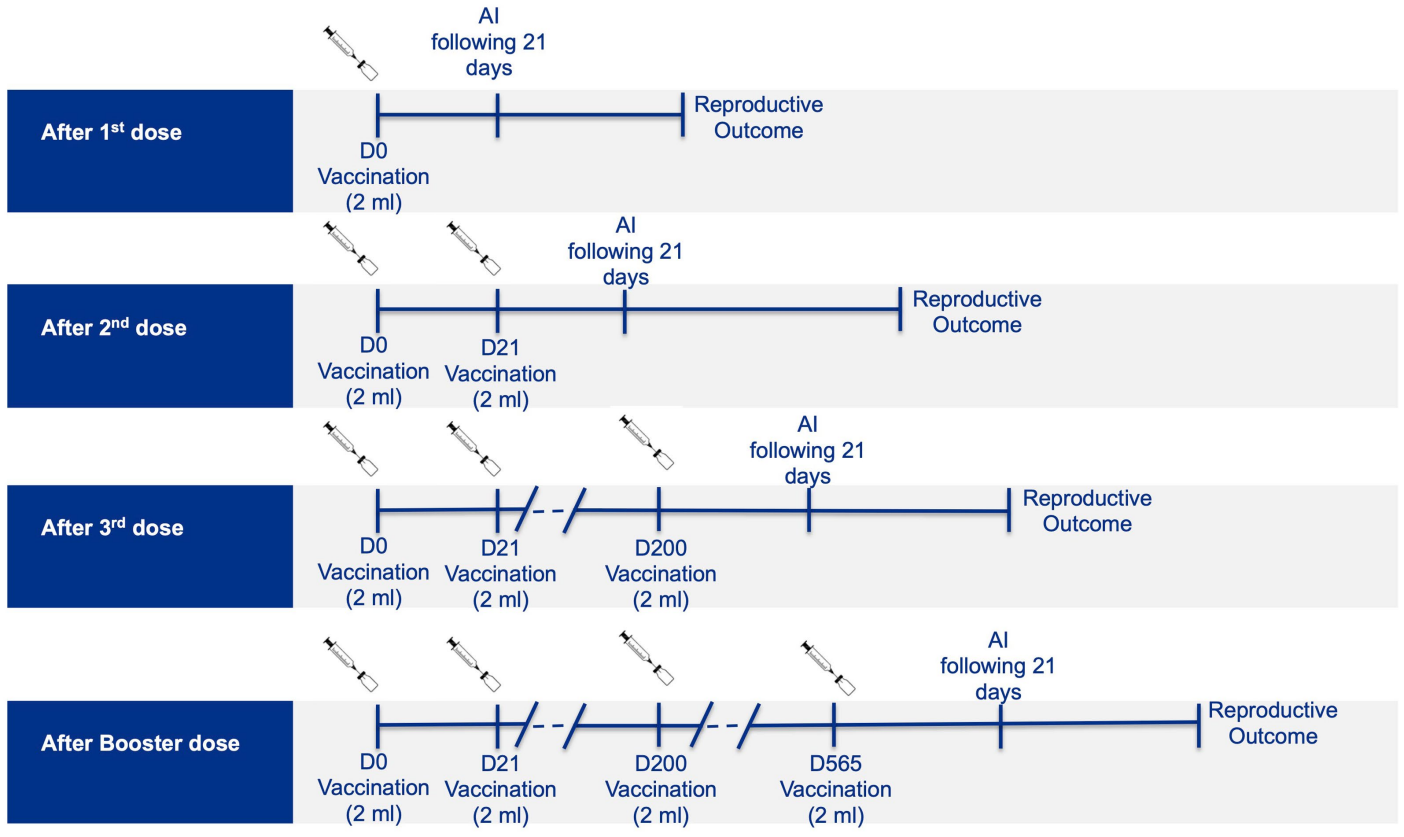
Design of field safety study in four herds.

##### 2.2.4.2 Pregnancy monitoring and reproductive performance recording

Pregnancy monitoring was performed through regular veterinary visits conducted by the designated farm veterinarian or the investigator, as per standard practice on dairy farms. These visits consisted of a pregnancy diagnosis on days 28–40 after service and successive visits to monitor pregnancy status around 50–90 and 200 days after service. During these follow-up visits, the investigator recorded the pregnancy losses found. In addition, the farmer/farm personnel observed daily all pregnant animals to detect any pregnancy loss (early embryonic death/abortions/stillbirth).

Reproductive performance was measured by assessing conception rate and pregnancy losses. Conception rate was calculated as the proportion of positive services (confirmed pregnancies) out of the total number of services performed during the 21 days following each vaccination dose. This timeframe was chosen to align with the vaccine administration schedule and to ensure that any potential impact on fertility was captured. Pregnancy losses were calculated as the total number of early embryonic deaths, abortions, and stillbirths versus the total number of animals that correctly received the re-vaccination schedule (1st, 2nd, and 3rd dose).

### 2.3 Statistical analysis

Sample size for preclinical study 1 was defined in accordance with the principles of the 3Rs (Replacement, Reduction, and Refinement). The study design followed well-established BoHV-1 latency/reactivation models, in which small group sizes are commonly used and considered sufficient to detect viral recrudescence following dexamethasone treatment (4). For the remaining preclinical studies, the sample size was defined in accordance with the European Pharmacopoeia (Monograph 1952; 04/2013: Bovine Viral Diarrhoea vaccine), which specifies minimum group sizes of 13 vaccinated and 7 control animals for efficacy studies, and at least 8 animals per group for safety studies. In the present study, group sizes exceeded these minimum requirements. For the field study, sample size was estimated based on simulations of the expected results for the primary efficacy variable (incidence of persistently infected animals), using Ene 3.0 software (Universitat Autònoma de Barcelona, Barcelona, Spain) and data from previous studies. Expected values of 2% for the control group and 0.02% for the vaccinated group were assumed. To achieve a statistical power of 80% to detect differences under the null hypothesis (*H*_0_: *μ*1 = *μ*2) for two independent samples, with a significance level of 5%, and allowing for an expected drop-out rate of 15%, it was determined that at least 471 animals per group were required.

Categorical variables were presented as frequencies and percentages, and quantitative variables as means and standard deviations (SDs) to summarize the general reproductive incidence. For the preclinical studies, reproductive performance parameters were compared between groups using a Fisher’s exact test (conception rate). For the field study, conception rate was evaluated using a logistic regression model, including group x dose interaction, along with category (heifer or cow) and lactation group (0, 1, 2, 3, or 4+) as fixed effects. Pregnancy loss rate was analysed using a mixed-effects logistic regression model, including the same fixed effects, with the animal nested within farm as a random effect. Among animals that experienced pregnancy loss, the number of days to abortion was analysed using a linear mixed-effects model with the same effects structure. For all statistical tests, a nominal significance level of 5% (*p-*value < 0.05) was applied. The statistical analysis was performed using R (version 4.2.2; R Core Team 2022; R Foundation for Statistical Computing, Vienna, Austria).

## 3 Results

### 3.1 Study 1: BoHV-1 latency reactivation and re-excretion after vaccination and dexamethasone treatment

#### 3.1.1 Intranasal virus shedding

The BoHV-1-positive control group began shedding BoHV-1, 3 days post-dexamethasone treatment (Study day 94), when 2 of 4 animals (50%) tested positive (Table 4). The proportion of positive control animal shedding increased to 100% (4 of 4) on days 7 and 8 post-dexamethasone treatment (Study days 97–98; Table 4). Shedding subsequently decreased, and by day 13 post-dexamethasone treatment (Study day 103), only 1 of 4 animals remained positive, marking the last day of detectable virus (1 of 4). The BoHV-1 shed by the control animals was evaluated by PCR, which was identical to the intranasally administered BoHV-1 Iowa Strain (data not shown). At no point during the study did any of the BoHV-1 (gE−/tk−) vaccinated animals shed virus (Table 4). Interestingly, one of the negative-control animals, which was commingled with the BoHV-1-positive animals from Study day 91 onwards (following the challenge phase), began shedding virus on day 13 post-dexamethasone treatment (Study day 103) and continued through the end of the nasal collections at day 105 (Table 4).

**Table 4:** Recrudescence following dexamethasone treatment (% BoHV-1 shedding; positive/total).

Group	Dexamethasone treatment					Days post vaccination/BHV-1 challenge										
	D91	D92	D93	D94	D95	D96	D97	D98	D99	D100	D101	D102	D103	D104	D105	D111^*^
Vaccine	0% (0/4)	0% (0/4)	0% (0/4)	0% (0/4)	0% (0/4)	0% (0/4)	0% (0/4)	0% (0/4)	0% (0/4)	0% (0/4)	0% (0/4)	0% (0/4)	0% (0/4)	0% (0/4)	0% (0/4)	0% (0/4)
BHV-1 positive control	0% (0/4)	0% (0/4)	0% (0/4)	50% (2/4)	75% (3/4)	75% (3/4)	100% (4/4)	100% (4/4)	100% (4/4)	75% (3/4)	25% (1/4)	25% (1/4)	25% (1/4)	0% (0/4)	0% (0/4)	100% (4/4)
Negative control	0% (0/2)	0% (0/2)	0% (0/2)	0% (0/2)	0% (0/2)	0% (0/2)	0% (0/2)	0% (0/2)	0% (0/2)	0% (0/2)	0% (0/2)	0% (0/2)	50% (1/2)	50% (1/2)	50% (1/2)	100% (2/2)

* Necropsy-PCR/VI positive trigeminal ganglion/tonsil.

#### 3.1.2 Serology

All positive control and vaccinated animals tested positive for BoHV-1 gB ELISA at day 91. In contrast, negative control animals remained negative at this time point (data not shown).

#### 3.1.3 Necropsy samples

All animals were humanely euthanized, necropsied, and the trigeminal ganglion and palatine tonsil were collected on day 111 (day 21 post-dexamethasone treatment) for BoHV-1 PCR testing (Table 4). All positive and negative (co-mingled) control animals were BoHV-1 PCR-positive. The BoHV-1 (gE−/tk−) vaccinated animals were all PCR-negative.

### 3.2 Study 2: preclinical effect of vaccination timing on conception rate

#### 3.2.1 Heifer calves (trials A and B)

In Trial A, the conception rate was similar between groups, reaching 75% in vaccinated animals and 80% in unvaccinated animals (Table 5).

**Table 5:** Conception rate of preclinical trials.

Study	Group	Number of animals	Days of vaccination	Day of AI	Days AI after vaccination	Pregnant after 1st AI	Days after vaccination of 2nd AI	Pregnant after 2nd AI	Pregnant after 1st and 2nd AI (conception rate)
Trial A	Vaccine	20	D0, D21 and D203	D260	57	15 (75%)	–	–	15 (75%)
	Control	10				8 (80%)			8 (80%)
					*p*-value^*^	1.00			
Trial B	Vaccine	20	D0, D21 and D203	D262	59	11 (55%)	94	6 of 9 (67%)	17 (85%)
	Control	10				5 (50%)		1 of 5 (20%)	6 (60%)
					*p*-value^*^	1.00		0.266	0.181
Trial C	Vaccine	20	D0 and D21	D43	22	13 (65%)	78	2 of 7 (29%)	15 (75%)
	Control	10				6 (60%)		1 of 4 (25%)	7 (70%)
					*p*-value^*^	1.00		1.00	1.00
Trial D	Vaccine	20	D0 and D21	D44	23	13 (65%)	86	3 of 7 (43%)	16 (80%)
	Control	10				6 (60%)		1 of 4 (25%)	7 (70%)
					*p*-value^*^	1.00		1.00	0.657

* Fisher’s exact test.

In Trial B, following the first breeding, the conception rates were comparable between groups (55% in the vaccinated group vs. 50% in the control group; Table 5). Of the remaining open heifers, 9 of the 11 vaccinated heifers and all five control open heifers were synchronized and rebred at 94 days (Table 5). Six of 9 (67%) vaccinated rebred heifers were pregnant, resulting in a conception rate of 85% among vaccinated heifers. This compares with only 1 of 5 rebred control heifers becoming pregnant, yielding a total conception rate of 60% in control animals (Table 5).

#### 3.2.2 Yearling heifers (trials C and D)

In Trial C, following the first breeding, the conception rates were similar between groups (65% in the vaccinated group vs. 60% in the control group; Table 5). Of the remaining open heifers, all seven open, vaccinated heifers, and all four control open heifers were synchronized and rebred at 94 days (Table 5). Two of 7 (29%) of the vaccinated rebred heifers were pregnant, resulting in a total of 75% conception rate for the vaccinated heifers, compared with only 1 of 4 rebred control heifers becoming pregnant, yielding a total conception rate of 70% in control animals (Table 5). In Trial D, following the first breeding, the conception rates were also comparable between groups (65% in the vaccinated group vs. 60% in the control group; Table 5). Of the remaining open heifers, all seven open, vaccinated heifers, and all four control open heifers were synchronized and rebred at 94 days (Table 5). Three of 7 (43%) of the vaccinated rebred heifers were pregnant, resulting in a total of 80% conception rate for the vaccinated heifers. This compared with only 1 of 4 rebred control heifers becoming pregnant, yielding a total conception rate of 70% in control animals (Table 5).

Overall, vaccination did not adversely affect conception rate across trials.

### 3.3 Study 3: preclinical effect of vaccination of animals during gestation

#### 3.3.1 Trial 1—effect of vaccination with the field vaccine dose during gestation

There were no abortions at any stage of gestation, and all calves were alive at parturition (Table 6). However, there were obstetric difficulties with one calf in the second-trimester group and one calf in the third-trimester group, resulting in both of the calves dying of hypoxia during delivery. In addition, one calf in the second trimester group was born alive but died following birth in an accidental feeder-related incident (Table 6). All remaining calves were normal following birth, and none of the calves were born with BoHV-1 precolostral antibodies (Table 6).

**Table 6:** Reproductive outcome and calf health following gestational vaccination.

Gestational age	Abortions	% Born live	Neonatal clinical signs (days postnatal)				Precolostral BoHV-1 antibodies positive
			Day 0	Day 1	Day 2	Day 3	
1st trimester	0% (0/9)	100% (9/9)	0% (0/9)	0% (0/9)	0% (0/9)	0% (0/9)	0% (0/9)
2nd trimester	0% (0/9)	100% (9/9^*^)	0% (0/7)	0% (0/7)	0% (0/7)	0% (0/7)	0% (0/7)
3rd trimester	0% (0/10)	100% (10/10^#^)	0% (9/9)	0% (9/9)	0% (9/9)	0% (9/9)	0% (0/9)

* 2 calves died at/following parturition-1 hypoxia during parturition; 1 born live but died stuck on a feeder.

# 1 calf died of hypoxia during parturition.

#### 3.3.2 Trial 2—effect of vaccination with a 10-fold (10X) increase of the IBR antigen content during pregnancy

There were no abortions at any stage of pregnancy, and all calves were born alive (Table 7). In addition, all calves were normal following birth, and none of the calves were born with BoHV-1 precolostral antibodies (Table 7).

**Table 7:** Reproductive outcome and calf health following gestational 10X BoHV-1 antigen vaccination.

Gestational age	Abortions	% born live	Neonatal clinical signs (days postnatal)				Precolostral BHV-1 antibodies positive
			Day 0	Day 1	Day 2	Day 3	
4 months	0% (0/9)	100% (9/9)	0% (0/9)	0% (0/9)	0% (0/9)	0% (0/9)	0% (0/9)
5 months^*^	0% (0/8)	100% (8/8)	0% (0/8)	0% (0/8)	0% (0/8)	0% (0/8)	0% (0/8)
6–7 months	0% (0/10)	100% (10/10)	0% (0/10)	0% (0/10)	0% (0/10)	0% (0/10)	0% (0/10)

* One heifer was originally in the 5-month group, but her gestation length belonged in the 6–7-month group.

### 3.4 Study 4: field study of the effect of vaccination on reproductive safety

The conception rate was measured on one of the four farms (Farm 1) included in the study (Table 8). After the 1st, 2nd, and 3rd doses of the vaccine, conception rates were ~20% higher in the vaccinated animals than in the controls. A statistically significant difference was observed after the 2nd dose (*p-*value = 0.025), whereas conception rates were similar between groups following the booster dose (Table 8).

**Table 8:** Conception rate for farm 1.

Vaccination dose	Control group			Vaccine group			*p*-value^#^
	Total services^*^	Positive services	Conception rate %	Total services^*^	Positive services	Conception rate %	
After 1st dose	29	4	13.8	24	9	37.5	0.053
After 2nd dose	43	7	16.3	39	14	35.9	0.025
After 3rd dose	29	10	34.5	20	11	55	0.205
After booster dose	15	8	53.3	15	7	46.7	0.991

* Only farm 1 results were available.

# Logistic regression model including group x dose interaction, category, and lactation group as fixed effects.

Pregnancy loss was assessed on all four farms (Table 9). Following each of the four vaccinations, pregnancy loss was comparable between the vaccinated and control animals. After the 1st vaccination, the pregnancy loss was similar in both groups. Although the vaccinated group showed a lower percentage of pregnancy loss following the 2nd, 3rd, and booster vaccinations, none of the differences were statistically significant. Overall, no adverse effect of vaccination on reproductive loss was observed.

**Table 9:** Pregnancy loss following vaccination for all farms.

Vaccination dose	Group	Vaccinated animals at each dose	Number lost	%	*p*-value^*^	Mean number of days	*p*-value^#^	Earliest day	Latest day
After 1st dose	Control	624	5	0.8%	0.785	13	0.890	5	20
	Vaccine	631	6	1.0%		18		12	20
After 2nd dose	Control	617	22	3.6%	0.111	65	0.576	2	181
	Vaccine	623	15	2.4%		78		4	167
After 3rd dose	Control	529	46	8.7%	0.867	196	0.472	14	370
	Vaccine	550	46	8.4%		195		14	370
After booster dose	Control	417	21	5.0%	0.237	89	0.928	19	151
	Vaccine	410	14	3.4%		104		26	152

* Mixed-effects logistic regression model including group x dose, category, and lactation group as fixed effects, and animal nested within farm as random effect.

# Mixed-effects linear regression model including group x dose, category, and lactation group as fixed effects, and animal nested within farm as random effect (only animals with pregnancy loss were included).

## 4 Discussion

These studies demonstrated the reproductive safety of a multivalent vaccine whose formulation includes the key reproductive immunogens BoHV-1 and BVDV, along with PI3 and BRSV. The vaccine did not establish latency, had no effect on conception rate regardless of the timing of administration throughout the reproductive cycle, and did not affect reproductive loss in pregnant animals. Unlike ordinary multivalent vaccines containing modified live BoHV-1 and BVDV, which result in reproductive loss (2, 4, 5), this vaccine has a safe reproductive profile.

The latency of bovine herpesvirus following infection or vaccination is well established as a mechanism driving long-term persistence and transmission within herds (4, 6, 18). Latency occurs with all ordinary modified live BoHV-1 vaccines (4), including temperature-sensitive vaccine virus such as RLB 106 (19), tk− mutant (20), gE− mutant (21), and quadruple mutant gE−, gG−, US9, and UL49.5 (22). In contrast, inactivated vaccines do not establish latency (4). Classically, latency and recrudescence are induced in animals following chemical immunosuppression with dexamethasone (18), a standard method applied in the present study. Notably, the lack of latency and recrudescence following dexamethasone administration in animals vaccinated with the MLV gE−/tk− BoHV-1 vaccine is novel. From a reproductive standpoint, the demonstration that progesterone can induce BoHV-1 reactivation during the reproductive cycle and heat stress (23) highlights the biological relevance of preventing BoHV-1 latency, given the occurrence of these “natural” stressors in cattle. The failure to demonstrate viral replication following dexamethasone treatment not only in the nasal cavity and trigeminal ganglion but also in the palatine/pharyngeal tonsil is also relevant. In cattle, the pharyngeal tonsil is a major site for the production of BoHV-1 following reactivation and is a key reservoir that facilitates transmission of reactivated BoHV-1 (6).

The adverse effects of MLV BoHV-1 vaccination on ovarian function and reproductive performance in non-pregnant females are well established (2–5, 24, 25). Conception rates can be dramatically decreased in naïve heifers following MLV BoHV-1/BVDV vaccination prior to breeding (2), and declines of 5–9% have also been reported in previously vaccinated animals receiving an additional MLV BoHV-1/BVDV dose (3, 5). This study evaluated the effect of vaccination on conception rate through both preclinical and field trials. Four preclinical trials were conducted in two different age groups, young calves (2–4 months) and yearling heifers. Across all trials, vaccination did not adversely affect conception rate, with no consistent differences observed between vaccinated and control animals. Vaccination during heifer development, with subsequent revaccination 6 months later, had no adverse effect on conception. Likewise, in the two trials involving previously immunized yearling heifers, administration of the vaccine 22 or 23 days prior to AI breeding had no effect on conception. These results are in contrast with those reported in an extensive field study with previously vaccinated animals (5), in which administration of conventional BoHV-1/BVDV MLV vaccines at 37 days or less prior to AI breeding resulted in an 8% decrease in AI conception rates. Consistent results were also observed in the field trial, where all animals were vaccinated simultaneously (mass vaccination), including a substantial proportion that had already been inseminated. Although conception data were available from only one participating farm, the vaccinated animals showed a significantly higher conception rate than controls after the second dose, with no evidence of adverse effects on reproductive performance, supporting the favorable reproductive safety profile of the vaccine.

Although most of the effects on the reproductive cycle and the ovary are related to BoHV-1, MLV BVDV vaccines also reach the ovaries and the developing follicles. Cytopathic BVDV has been isolated from the ovaries until day 12 post-vaccination, and BVDV antigen remains detectable up to 30 days post-vaccination, raising concerns that replication of virus in the ovary could cause ovarian dysfunction and reduced fertility (26). A meta-analysis of BVDV vaccination and reproductive outcomes demonstrated a 5% increase in pregnancy risk in cattle vaccinated with a MLV BVDV vaccine across 5 field trials. However, this increase in risk was not observed in controlled experimental studies (27). Given the large field studies that demonstrated decreased reproductive performance following MLV vaccination (3, 5), it is not possible to attribute the effects on conception and reproductive performance solely to MLV BoHV-1, as all the MLV vaccines used in those studies also contained MLV BVDV. In contrast, the multivalent vaccine used in the present study contains a recombinant BVDV E2 glycoprotein that cannot undergo viral replication in ovarian tissue and therefore provides a broader safety profile.

The other major safety reproductive parameter is vaccination of pregnant animals. The ability of ordinary MLV BoHV-1 vaccines to induce abortion storms in BoHV-1 naïve pregnant females is well established, with reported abortion rates ranging from 20 to 50% in susceptible herds (4). Interestingly, even BoHV-1 tk− mutant induced abortions in 2 of 5 inoculated pregnant heifers (28). MLV BoHV-1 vaccines, however, have been effective at reducing the overall incidence of BoHV-1 associated abortion rates from 16% in the 1960s (29) to less than 2.0% (1.4–2.8%) in 2007–2013 (30). Because of the low incidence of BoHV-1 abortions and the high use of conventional MLV BoHV-1 vaccines, more than 95% of BoHV-1-positive abortion submissions since ~2009 in US diagnostic laboratories have been attributed to vaccine strains rather than wild-type virus (4). Although such vaccine-associated cases occur, the absolute number remains relatively low to the millions of doses administered each year. Thus, if “safe” is defined as “rarely associated with abortion,” current MLV vaccines meet that criterion; however, if “safe” implies “never associated with abortion,” they do not (4).

In line with these considerations, another often-overlooked safety aspect is the vaccination of nursing calves with ordinary MLV IBR/BVD vaccines when they are in close contact with their pregnant dams. The need for early protection against major respiratory viruses is well recognized, given their high prevalence, including IBR, BRSV, and BVDV (31). Several commercial MLV vaccines explicitly advise against vaccinating calves that are nursing pregnant cows, due to the potential risk of transmission of vaccine virus to pregnant females, which can result in abortion.

To address these concerns, the present study evaluated pregnancy safety under both controlled and field conditions. Across the trimester-based assessment using standard field dose and the high-dose evaluation (10X field vaccine dose) conducted at 4, 5, or 6 months of gestation, no abortions were observed. All live calves in these studies were BoHV-1 seronegative at birth, and the neonatal calves were healthy, with no clinical signs. An earlier preclinical study using a similar dosing design and BoHV-1 strain as in the multivalent vaccine, also reported no abortions and healthy BoHV-1 seronegative calves at birth (17). The results from the field study are consistent with these observations, all animals on each farm were vaccinated at the same time, with multiple doses, including a large number in the 1st, 2nd, and 3rd trimesters. There was no difference between vaccinated and control animals for any of the reproductive outcomes assessed.

## 5 Conclusion

In conclusion, these studies demonstrated that the novel gene-deleted BoHV-1 strain (gE−/tk−) used alone or in a multivalent formulation containing recombinant BVDV1 and 2 E2 proteins, live-attenuated BRSV, and inactivated PI3 has a strong reproductive safety profile. Unlike ordinary MLV BoHV-1 vaccines, the gE−/tk− formulation showed no evidence of latency, viral reactivation, reduced conception, or increased pregnancy loss. Overall, the results consistently support the safe use of the vaccine in both heifers and pregnant cattle.

## Data Availability

The datasets generated and analyzed for this study are available from the corresponding author upon reasonable request.

## References

[1] Newcomer BW Givens MD. Diagnosis and control of viral diseases of reproductive importance. Vet Clin North Am Food Anim Pract. (2016) 32:425–41. doi: 10.1016/j.cvfa.2016.01.011, 27140298

[2] Perry GA Zimmerman AD Daly RF Buterbaugh RE Rhoades J Scholz D . The effects of vaccination on serum hormone concentrations and conception rates in synchronized naive beef heifers. Theriogenology. (2013) 79:200–5. doi: 10.1016/j.theriogenology.2012.10.005, 23127919

[3] Perry GA Larimore EL Crosswhite MR Neville BW Cortese VS Daly RF . Safety of vaccination with an inactivated or modified live viral reproductive vaccine when compared to sterile saline in beef cows. Jacobs J Vet Sci Res. (2016) 2:1–7.

[4] Chase CCL Fulton RW O’Toole D Gillette B Daly RF Perry G. Bovine herpesvirus 1 modified live virus vaccines for cattle reproduction: balancing protection with undesired effects. Vet Microbiol. (2017) 206:69–77. doi: 10.1016/j.vetmic.2017.03.016, 28377131

[5] Perry GA Geary TW Walker JA Rich JJJ Northup EJ Perkins SD . Influence of vaccination with a combined chemically altered/inactivated BHV-1/BVD vaccine or a modified-live BHV-1/BVD vaccine on reproductive performance in beef cows and heifers. Bovine Pract. (2018) 52:53–8. doi: 10.21423/bovine-vol52no1p53-58

[6] Ostler JB Jones C. The bovine herpesvirus 1 latency-reactivation cycle, a chronic problem in the cattle industry. Viruses. (2023) 15:552. doi: 10.3390/v15020552, 36851767 PMC9966457

[7] Kit S Qavi H Gaines JD. Thymidine kinase-negative bovine herpesvirus type 1 mutant is stable and highly attenuated in calves. Arch Virol. (1985) 86:63–83. doi: 10.1007/BF01314114, 2994602

[8] Petrini S Iscaro C Righi C. Antibody responses to bovine alphaherpesvirus 1 (BoHV-1) in passively immunized calves. Viruses. (2019) 11:23. doi: 10.3390/v11010023, 30609738 PMC6356344

[9] Kaashoek MJ van Engelenburg FAC Moerman A Gielkens ALJ Rijsewijk FAM van Oirschot JT. Virulence and immunogenicity in calves of thymidine kinase- and glycoprotein E-negative bovine herpesvirus 1 mutants. Vet Microbiol. (1996) 48:143–53. doi: 10.1016/0378-1135(95)00137-9, 8701570

[10] van Engelenburg FAC Kaashoek MJ Rijsewijk FAM van den Burg L Moerman A Gielkens ALJ . A glycoprotein E deletion mutant of bovine herpesvirus 1 is avirulent in calves. J Gen Virol. (1994) 75:2311–8. doi: 10.1099/0022-1317-75-9-2311, 8077929

[11] Tapiolas M Gibert M Montbrau C Taberner E Solé M Santo-Tomás H . Efficacy of a new multivalent vaccine for the control of bovine respiratory disease (BRD) in a randomized clinical trial in commercial fattening units. Vaccine. (2024) 12:1233. doi: 10.3390/vaccines12111233 PMC11598617 39591136

[12] Montbrau C Gibert M Solé M Barril I Roca M Acal L. Respiratory efficacy of a multivalent marker vaccine against bovine viral diarrhoea virus types 1 and 2, infectious bovine rhinotracheitis virus, bovine respiratory syncytial virus, and bovine parainfluenza-3 virus in young calves. Vaccine. (2025) 13:999. doi: 10.3390/vaccines13100999, 41150388 PMC12568246

[13] Taberner E Gibert M Montbrau C Ruiz IM Mallorquí J Santo-Tomás H . Efficacy of vaccination with the DIVENCE® vaccine against bovine viral diarrhea virus types 1 and 2 in terms of fetal protection. Vet Med Res Rep. (2024) 15:221–38. doi: 10.2147/vmrr.s474655 PMC11645965 39679301

[14] Pacheco-Lima J Silva H Beneitez JPC da Silva DF Silva FM. Effect of vaccination against IBR/BVD on the reproductive performances of Brava dos Açores - a bovine Lidia breed. Am J Biomed Sci Res. (2019) 6:266–72. doi: 10.34297/ajbsr.2019.06.001041

[15] Duffy R Gavin T Couper A Quille J McLaughlin D Hanly E . Evolution of bovine herpesvirus-1 infection prevalence and infection dynamics in Irish dairy herds following an IBR hyperimmunisation vaccination protocol. Ir Vet J. (2025) 79:10. doi: 10.1186/s13620-025-00328-w, 41437296 PMC12853841

[16] WOAH. "Infectious bovine rhinotracheitis/infectious pustular vulvovaginitis". In: WOAH terrestrial manual, chapter 3.4.11 Paris, France: World Organzation of Animal Health (2024)

[17] Moreno A Desmiquels E Casado C Roca M. "Safety of a new attenuated IBR live vaccine with double genetic deletion gE−tk−: dissemination, latency/re-excretion and abortigenicity: three trials assaying very high doses". In: Proceedings of the 27th World Buiatrics Congress. Lisbon, Portugal: World Buiatrics Congress (2012). p. 222.

[18] El-Mayet F Jones C. Stress can induce bovine alpha-herpesvirus 1 (BoHV-1) reactivation from latency. Viruses. (2024) 16:1675. doi: 10.3390/v16111675, 39599791 PMC11599084

[19] Jones C Newby TJ Holt T. Analysis of latency in cattle after inoculation with a temperature sensitive mutant of bovine herpesvirus 1 (RLB106). Vaccine. (2000) 18:3185–95. doi: 10.1016/S0264-410X(00)00106-7, 10856798

[20] Whetstone CA Miller JM Seal BS Bello LJ Lawrence WC. Latency and reactivation of a thymidine kinase-negative bovine herpesvirus 1 deletion mutant. Arch Virol. (1992) 122:207–14. doi: 10.1007/BF01321129, 1309641

[21] Mars MH de Jong MCM van Oirschot JT. A gE-negative bovine herpesvirus 1 vaccine strain is not re-excreted nor transmitted in an experimental cattle population after corticosteroid treatments. Vaccine. (2000) 18:1975–81. doi: 10.1016/S0264-410X(99)00536-8, 10706958

[22] Pavulraj S Stout RW Paulsen DB Chowdhury SI. A quadruple gene-deleted live BoHV-1 subunit RVFV vaccine vector reactivates from latency and replicates in TG neurons of calves but is not transported to or shed from nasal mucosa. Viruses. (2024) 16:1497. doi: 10.3390/v16091497, 39339973 PMC11437494

[23] El-Mayet FS Toomer G Ostler JB Harrison KS Santos VC Wijesekera N . Progesterone sporadically induces reactivation from latency in female calves but proficiently stimulates bovine herpesvirus 1 productive infection. J Virol. (2022) 96:e0213021-21. doi: 10.1128/jvi.02130-21, 35019726 PMC8906428

[24] Miller JM van der Maaten MJ Whetstone CA. Infertility in heifers inoculated with modified-live bovine herpesvirus-1 vaccinal strains against infectious bovine rhinotracheitis on post-breeding day 14. Am J Vet Res. (1989) 50:551–4. doi: 10.2460/ajvr.1989.50.04.551, 2540687

[25] Epperson KM Rich JJJ Zoca SM Quail LK Andrews TN Kline AC . Influence of commercial inactivated or modified-live virus vaccination at time of AI on corpus luteum development and function in beef cattle. Anim Reprod Sci. (2024) 270:107594. doi: 10.1016/j.anireprosci.2024.107594, 39236590

[26] Grooms DL Brock KV Ward LA. Detection of cytopathic bovine viral diarrhea virus in the ovaries of cattle following immunization with a modified live bovine viral diarrhea virus vaccine. J Vet Diagn Invest. (1998) 10:130–4. doi: 10.1177/10406387980100020, 9576338

[27] Newcomer BW Walz PH Givens MD. Efficacy of bovine viral diarrhea virus vaccination to prevent reproductive disease: a meta-analysis. Theriogenology. (2015) 83:360–5.e1. doi: 10.1016/j.theriogenology.2014.09.028, 25447148

[28] Miller JM Whetstone CA Bello LJ Lawrence WC Whitbeck JC. Abortion in heifers inoculated with a thymidine kinase-negative recombinant of bovine herpesvirus 1. Am J Vet Res. (1995) 56:870–4. doi: 10.2460/ajvr.1995.56.07.870 7574153

[29] Kirkbride CA Bicknell EJ Reed DE Robl MG Knudtson WU Wohlgemuth K. A diagnostic survey of bovine abortion and stillbirth in the Northern Plains states. J Am Vet Med Assoc. (1973) 162:556–60. doi: 10.2460/javma.1973.162.07.556, 4692821

[30] South Dakota Animal Disease Research and Diagnostic Laboratory (SDADRDL). Annual Reports. 2008, 2013 and 2014. Brookings, SD USA: Animal Disease Research Laboratory, Brookings SD USA (2007).

[31] Santo-Tomás H Barreto M Vazquez B Villoria P Teixeira R Solé M. Bovine respiratory disease complex: prevalence of the main respiratory viruses involved in pneumonia in Spain. J Anim Sci Res. (2023) 7:1–5. doi: 10.16966/2576-6457.163

